# Abandonment of prescriptions in medically underserved areas: Primary medication non-adherence in community pharmacies in the delta region of the United States

**DOI:** 10.1016/j.rcsop.2024.100484

**Published:** 2024-07-28

**Authors:** Minghui Li, Matthew Harmon, Mike Wasson, Lindsey Cardosi, Lindsey Henson, Hunter Hill, Brad Ian Jobe, Sydnee E. Hewitt, Kenneth C. Hohmeier

**Affiliations:** a University of Tennessee Health Science Center, College of Pharmacy, Nashville, TN, United States of America; b Kroger Pharmacy, Memphis, TN, United States of America

**Keywords:** Community pharmacy services, Pharmacies, Medically underserved area, Medication adherence

## Abstract

**Background:**

In the U.S. alone, medication non-adherence is estimated to cause 1 in 10 hospitalizations, approximately 125,000 deaths annually, and cost the U.S. healthcare system just under $300 billion each year. Patients in medically underserved areas (MUAs) are particularly vulnerable to all forms of non-adherence and downstream morbidity and mortality; however, the extent to which primary medication non-adherence (i.e., prescription abandonment) affects the underserved is still largely unknown.

**Objectives:**

To assess the difference in rates of abandonment of quality measured prescriptions in areas that are medically underserved compared to areas that are not. The secondary objective is to assess the impact that the COVID-19 pandemic had on rates of prescription abandonment in both MUAs and those that are not.

**Methods:**

In this retrospective study, data on abandoned, quality measured prescriptions were collected and analyzed using Chi-Square analyses from one regional division of a large grocery-chain pharmacy containing ninety-one pharmacies located in Tennessee, Mississippi, Arkansas, Kentucky, and Missouri. The primary objective used 2019 data while the secondary objective used data from April – November of both 2019 and 2020.

**Results:**

Patients from MUAs abandoned quality measured prescriptions at a higher rate of 5.44% compared to 4.77% of those not living in these areas (*P* < 0.01). This study also discovered that during the COVID-19 pandemic, MUAs had a decrease in abandonment from 6.14% in 2019 to 6.02% in 2020 (*P* < 0.01). Those from non-MUAs had non-significant change in abandonment (*P* = 0.87).

**Conclusion:**

Patients in MUAs abandon quality measured prescriptions at a statistically significant higher rate when compared to patients who live in areas that are not considered to be medically underserved. Moreover, during the COVID-19 pandemic patients living in MUAs had a statistically significant decrease in prescription abandonment while those in non-MUAs did not statistically change.

## 1 Background

Prescription abandonment is the phenomenon where a prescription order is sent to the pharmacy (electronically, telephonically, or facsimile) but is never picked up by the patient, and it is a well documented form of medication non-adherence.^1^ In the U.S. alone, medication non-adherence is estimated to cause 1 in 10 hospitalizations, approximately 125,000 deaths annually, and cost the U.S. healthcare system just under $300 billion each year.^2^, ^3^, ^4^

There are several factors associated with prescription abandonment. Some of the most common of these include high copay,^1^^,^^5^, ^6^, ^7^, ^8^, ^9^, ^10^, ^11^, ^12^, ^13^, ^14^, ^15^, ^16^ being an electronic prescription,^1^^,^^17^ and complex medication regimens.^18^ Excluding specialty medications (i.e., high cost medications used to treat rare or complex conditions), the prescription abandonment rate ranges between 1.7 and 13.9%.^1^^,^^19^, ^20^, ^21^, ^22^, ^23^, ^24^, ^25^, ^26^

Patients residing in medically underserved areas (MUAs) are particularly vulnerable to the negative consequences of medication non-adherence and its consequences for a multitude of reasons including a lack of healthcare providers and generally having lower-income as compared with non-MUA counterparts.^27^^,^^28^ MUAs are geographic locations that have been designated as such by the Health Resource & Services Administration given that there are too few primary care providers, high infant mortality, high poverty, or a high elderly population.^29^ Previous studies have shown that minorities, low-income earners, and elderly patients living in these areas generally have worse adherence rates when compared to those not in these groups.^30^, ^31^, ^32^, ^33^, ^34^, ^35^

Medication non-adherence has become a target for national quality improvement initiatives in the U.S.^36^ In an effort to improve the quality of healthcare delivery through tracking and incentivizing improved patient outcomes (i.e., value-based care), the Centers for Medicare & Medicaid Services (CMS), for example, have endorsed several secondary adherence-based quality measures for diabetes, hypertension, and cholesterol to improve care and reduce healthcare costs for their beneficiaries.^37^ And, the implementation of these CMS quality measures have led to significant increases in secondary medication adherence across all three disease states.^38^ However, these quality measures are centered on secondary medication adherence (i.e., continuing adherence after an initial fill has been picked up by a patient) rather than primary medication non-adherence, whereby a patient never picks up the initial fill of a prescribed medication.^37^

Despite the growing attention being placed on pharmacist interventions to improve medication adherence, most efforts have centered solely on secondary medication non-adherence.^26^ Although much is now known about the causes, impact, and successful interventions to address secondary non-adherence, primary medication non-adherence due to prescription abandonment has gone largely unexplored in previous research. Moreover, because of the unique socioeconomic makeup of MUAs, these communities may share a disproportionate negative impact of prescription abandonment given that cost and medication regimen complexity are correlated with higher abandonment rates.^1^^,^^5^, ^6^, ^7^, ^8^, ^9^, ^10^, ^11^, ^12^, ^13^, ^14^, ^15^, ^16^^,^^18^ Lastly, because of the timing of the study in the context of the COVID-19 pandemic, and because of the known disproportionate impact that COVID-19 had on MUAs, a secondary analysis was performed to investigate the impact of the pandemic on this population.

## 2 Objectives

The primary objective of this study is to compare rates of abandonment of quality measured prescriptions in MUAs as compared with non-MUAs prior to the COVID-19 pandemic. The secondary objective is to assess the impact that the COVID-19 pandemic had on rates of prescription abandonment in both MUAs and those that are not.

## 3 Methods

This was a retrospective study evaluating prescription abandonment rates using prescription data abstracted from pharmacy software reports from one regional division of a national grocery-chain pharmacy. The regional division included ninety-one pharmacies located in the U.S. Delta Region, across the U.S. states of Tennessee, Mississippi, Arkansas, Kentucky, and Missouri. Approval for this project was granted by the University of Tennessee Health Science Center Institutional Review Board on April 19, 2021.

For the primary objective, data ranged from January 2019 through December 2019. For the secondary objective, data ranged from April 2019 through November 2019 and April 2020 through November 2020. Abandonment rates were measured as the average rates during the study periods. These time periods for the secondary objective were chosen to assess the impact the COVID-19 pandemic may have had on prescription abandonment prior to emergency use authorization approved vaccines.

Data collected in the prescription abandonment reports included the variables of patient age, sex, zip code, automatic refill status, copay amount, and medication name and class. Patient age was measured in years and classified as children (0–17 years), young adults (18–44 years), middle-aged adults (45–64 years), and older adults (65 years and older). Patient sex was classified as female and male. To determine if the patient lived in a MUAs, patient zip codes were cross-referenced with data from the “Medically Underserved Area Tool” provided by the Health Resources and Services Administration.^29^ Automatic refill status was measured based on whether the patient received automatic refills for their prescriptions. Copay amount was measured in dollars and classified as $0, $0.01–$5, $5.01–$10, $10.01–$20, and more than $20. Quality measured prescription medications that were included in this study were those whose adherence rates are rated by the Centers for Medicare & Medicaid Services, which include oral anti-diabetic medications, renin-angiotensin-system antagonists, and HMG-CoA reductase inhibitors.^37^

Quality measured prescriptions were selected based on CMS endorsed Pharmacy Quality Alliance (PQA) measures. These medications include oral anti-diabetics,^13^ renin-angiotensin-system antagonists (RASAs),^11^ and HMG-CoA reductase inhibitors (statins).^15^^,^^28^^,^^36^ To appropriately analyze the data, variables containing continuous data were grouped together. This study used standard age groups in the analysis. Study participants were classified based on age as children (0–17), young adults (18–44), middle-aged adults (45–64), and old adults (65 and older). For patient copay cost, the five groups were $0, $0.01 – $5.00, $5.01 – $10.00, $10.01 – $20.00, and greater than $20.00.

Descriptive analyses were conducted to report patient characteristics in the overall sample and by MUA status. Chi-square tests were used to compare the rates of abandonment between those located in MUA and non-MUA areas and between pre- and post-pandemic periods. Bonferroni corrections were used to account for multiple comparisons, with *p*-values for significance testing adjusted accordingly by dividing 0.05 by the number of tests conducted. Logistic regression analyses were performed to identify factors associated with prescription abandonment by controlling for covariates including age, sex, automatic refill status, patient copayment, and dug class. The final model was derived using the backward elimination method, retaining all variables with *p*-values below 0.05. The effect was measured using odds ratios, accompanied by 95% confidence intervals. Missing data constituted <1% of all data and those observations were excluded. All analyses were conducted using SAS 9.4 (SAS Institute, Inc., Cary, NC).

## 4 Results

Complete patient demographics are found in Table 1. In total, 1,443,420 patients were included in the study. Most patients were in MUAs (*n* = 1,184,831; 61.8%) with an almost even distribution across sexes (male: *n* = 709,140; 49.13%). Patient copays varied across MUA as compared with non-MUA locations (*P* = 0.01), with most copays being less than $20 in both groups. Automatic refill services were used more often in non-MUA areas (MUA: *n* = 146,190; 12.34% versus non-MUA: *n* = 34,576; 13.47%; *P* < 0.01).

**Table 1:** Patient Demographics.

Characteristics	All		MUA		Non-MUA	
	n	%	n	%	n	%
Age, mean						
Male	709,140	49.13	578,574	48.83	129,454	50.45
Female	734,280	50.87	606,257	51.17	127,163	49.55
MUA	1,184,831	82.20				
Non-MUA	256,617	17.80				
Patient Copay						
$0	384,537	26.64	314,148	26.51	69,790	27.20
$0.01 - $5	422,117	29.24	348,779	29.44	72,786	28.36
$5.01 - $10	290,424	20.12	239,445	20.21	50,596	19.72
$10.01 - $20	225,734	15.64	185,057	15.62	40,353	15.72
$20+	120,608	8.36	97,402	8.22	23,092	9.00
Automatic Refill						
Yes	181,038	12.54	146,190	12.34	34,576	13.47
No	1,262,382	87.46	1,038,641	87.66	222,041	86.53
Drug Class						
RASA	571,074	39.56	467,062	39.42	103,229	40.23
Anti-diabetics	314,543	21.79	262,281	22.14	51,830	20.20
Statins	557,803	38.64	455,488	38.44	101,558	39.58

Quality measured prescriptions were abandoned significantly more frequently in MUAs than non-MUAs (5.44% vs. 4.77% (P < 0.01)) (Table 2). Table 3 summarizes the odds ratios for the different variables that could potentially influence the rate of abandonment and includes data from both the MUA and non-MUA groups. Groups most likely to abandon their prescriptions at the pharmacy were females, those aged 18–44, and those not enrolled in the pharmacy's automatic refill program. Increasing copayment amounts resulted in increasing odds for abandonment, with the highest rate of abandonment being for those with copayments greater than $20. The statin class of medications were most frequently abandoned, followed by anti-diabetic and renin-angiotensin-system antagonist medications.

**Table 2:** Abandonment Rates Between Medically Underserved Areas Compared with Non-Medically Underserved Areas.

Measure	MUA	Non-MUA	*P*-value
Rate of Abandonment (%)	5.44	4.77	*P* ≤0.0001
Odds Ratio, MUA vs non-MUA	(1.17, 95% CI: 1.142–1.199)

**Table 3:** Odds Ratios for Variables Associated with Prescription Abandonment, Combined.

Variable	Odds Ratio
Sex	
Female vs Male	(OR: 1.216, 95% CI: 1.194–1.238)
Patient Age Range	
0–17 vs 65+	(OR: 1.143, 95% CI: 0.933–1.399)
18–44 vs 65+	(OR: 1.801, 95% CI: 1.745–1.858)
45–64 vs 65+	(OR: 1.205, 95% CI: 1.181–1.229)
Automatic Refill	
Yes vs No	(OR: 2.257, 95% CI: 2.208–2.307)
Patient Copay	
$0.01 - $5 vs $0	(OR: 1.242, 95% CI: 1.207–1.277)
$5.01 - $10 vs $0	(OR: 1.397, 95% CI: 1.357–1.438)
$10.01 - $20 vs $0	(OR: 1.708, 95% CI: 1.657–1.761)
$20+ vs $0	(OR: 3.681, 95% CI: 3.569–3.796)
Drug Class	
Anti-diabetics vs RASA	(OR: 1.083, 95% CI: 1.054–1.112)
Statins vs RASA	(OR: 1.106, 95% CI: 1.083–1.13)

RASA = renin angiotensin system medication drug class.

Table 4 summarizes the odds ratios for the different variable groups across MUA or non-MUA cohorts. None of these differences between groups reached statistical significance. However, across all patient copay variables the MUA group had a higher odds of abandonment than the non-MUA group.

**Table 4:** Odds Ratios for Medically Underserved Areas Compared with Non-Medically Underserved Areas, Separated.

Variable	Odds Ratio, MUA	Odds Ratio, non-MUA
Sex		
Female vs Male	(OR: 1.22, 95% CI: 1.196–1.245)	(OR: 1.196, 95% CI: 1.144–1.252)
Patient Age Range		
0–17 vs 65+	(OR: 1.134, 95% CI: 0.909–1.415)	(OR: 1.186, 95% CI: 0.715–1.966)
18–44 vs 65+	(OR: 1.769, 95% CI: 1.71–1.831)	(OR: 1.977, 95% CI: 1.827–2.14)
45–64 vs 65+	(OR: 1.189, 95% CI: 1.163–1.215)	(OR: 1.29, 95% CI: 1.227–1.355)
Automatic Refill		
Yes vs No	(OR: 2.259, 95% CI: 2.205–2.314)	(OR: 2.246, 95% CI: 2.129–2.369)
Patient Copay		
$0.01 - $5 vs $0	(OR: 1.238, 95% CI: 1.2–1.276)	(OR: 1.257, 95% CI: 1.172–1.349)
$5.01 - $10 vs $0	(OR: 1.4, 95% CI: 1.356–1.445)	(OR: 1.38, 95% CI: 1.284–1.485)
$10.01 - $20 vs $0	(OR: 1.728, 95% CI: 1.672–1.787)	(OR: 1.603, 95% CI: 1.484–1.731)
$20+ vs $0	(OR: 3.762, 95% CI: 3.637–3.891)	(OR: 3.31, 95% CI: 3.069–3.569)
Drug Class		
Anti-diabetics vs RAS	(OR: 1.069, 95% CI: 1.038–1.101)	(OR: 1.15, 95% CI: 1.076–1.23)
Statins vs RAS	(OR: 1.111, 95% CI: 1.085–1.137)	(OR: 1.082, 95% CI: 1.026–1.141)

Table 5 describes the rates and odds ratios of quality measured prescription abandonment for MUAs and non-MUAs prior to and during the COVID-19 pandemic. For both MUA and non-MUA groups combined, the pre-pandemic rate of abandonment was higher than the rate during the pandemic (6.01% vs. 5.92%; *P* = 0.02). For the MUA group, the rate of abandonment decreased from 6.14% pre-pandemic to 6.02% during the pandemic (*P* < 0.01). When comparing the COVID-19 pandemic time period to the pre- pandemic time period, the odds ratio of abandonment in MUAs was 0.965 with a Wald's 95% confidence interval of 0.95–0.98. The rate of abandonment did not significantly change from the pre-pandemic to pandemic periods in the non-MUA group (*P* = 0.87).

**Table 5:** Abandonment Rates and Odds Ratios During COVID-19 Pandemic.

Group	Pre-Pandemic (%)	Pandemic (%)	*P*-value	Odds Ratio Pre-Pandemic vs Pandemic
MUA	6.14	6.02	*P* = 0.0073	(OR: 0.965, 95% CI: 0.95–0.98)
Non-MUA	5.43	5.45	*P* = 0.8692	(OR: 1.003, 95% CI: 0.969–1.038)
Combined	6.01	5.92	*P* = 0.0179	(OR: 0.971, 95% CI: 0.958–0.985)

**MUA =** Medically Underserved Areas Compared.

## 5 Discussion

The results of this study uncover a previously undocumented prescription access disparity between those patients living in MUAs as compared to those living elsewhere. Specifically, living in an MUA increases the patient's risk of abandoning their prescription at the pharmacy – contributing to the burden of medication non-adherence. Of note, these findings are particularly valuable because they add to our understanding of the under-researched area of primary medication non-adherence, or non-adherence related to starting a new medication.^26^

Despite having a higher overall rate of abandonment as compared with the non-MUA group, during the COVID-19 pandemic, unexpectedly, the MUA group had significantly lower rates of abandonment than pre-pandemic. There are several possible reasons for this. Given the “shelter in place” guidance promoted by government health authorities, the pharmacy chain in the study began proactively promoting a variety of adherence services to improve adherence. These included delivery of medications to the patient's home, medication automatic refill, and medication synchronization. Each of these interventions has an evidence-base in the literature as it relates to secondary medication adherence. Automatic refill services are well known to improve secondary medication adherence and reduce medication waste.^39^^,^^40^ Similarly, home delivery of medications has been shown to do the same when combined with other medication adherence services.^41^ Finally, a recent meta-analysis has found that medication synchronization, whereby medications are scheduled to be filled on the same day, also improved secondary medication adherence significantly.^42^ What is meaningful about this evidence base in light of the present study is that our analysis looked at primary medication non-adherence and, thefore, this may signal that these interventions may also improve primary, as well as secondary, non-adherence. Future prospective research will be needed to understand how and to what extent these medication adherence services impact primary medication non-adherence.

Overall, patients in MUAs had significantly higher rates of prescription abandonment than those in non-MUA. Given socioeconomic barriers found in MUAs, this is not surprising.^27^^,^^28^ Higher percentages of elderly persons and low-income earners are theoretically more sensitive to copay prices, and this was borne out in our analysis where those in the MUA group were more likely to abandon their prescription across every level of copay range. Although adherence services such as automatic refill, medication synchronization, and medication delivery may overcome many adherence barriers – it is unlikely that they will alone be sufficient to overcome cost-related issues. Recently, two studies investigated the impact of a primary medication non-adherence intervention targeting patient-specific barriers to adherence.^43^^,^^44^ Medication cost and “forgetfulness” were the two most common barriers to primary medication adherence.^44^ As previously discussed, services offered as part of the pharmacy chain's strategy to counter the COVID-era barriers to adherence likely impacted patient “forgetfulness.” However, Cason et al., also found that proactive pharmacist interventions aimed at initiating therapeutic interchange to lower cost medications and other copay lowering solutions significantly improved primary medication non-adherence.^44^

Another potential way to help decrease prescription abandonment specifically for MUAs is to increase health literacy. There is a strong correlation between health literacy and medication nonadherence, and overall there is a lower rate of health literacy amongst the medically underserved population.^45^ One solution may be to combine health literacy interventions within existing medication therapy management services. However, a challenge to this model is in the fact that individuals in urban areas are seven times more likely to complete a comprehensive medication review with their pharmacist or physician than those in rural areas.^46^ Increasing medication therapy management in MUAs may help decrease prescription abandonment or nonadherence rates, but how to target these populations to make these services accessible and utilized remains to be determined.

Our results found an overall range of prescription abandonment between patients living in an MUA and those who live in non-MUA regions between 5.44 and 4.77%. This is well within the range reported in the literature 1.7–13.9%.^1^^,^^14^, ^15^, ^16^, ^17^, ^18^, ^19^, ^20^, ^21^ However, accurately measuring prescription abandonment is substantially more challenging than that of secondary medication non-adherence for several reasons. First, prescription abandonment occurs when the “prescription is filled by a pharmacy but not claimed by the patient”.^36^ Given increasing rates of electronic prescribing, it is now possible to have a more complete view of what prescribed medications are never claimed, it is still an incomplete picture. Paper prescriptions which are never brought to the pharmacy represent one such gap in the picture, while the other gap is due to the fact that electronically generated prescriptions sent to a pharmacy but not filled will never have a corresponding insurance “claim”, and therefore may be missed by most studies using claims data to investigate medication adherence. New methods to consistently collect and report both primary and secondary non-adherence are needed, especially in light of the present study uncovering a primary medication non-adherence health disparity.

Another salient result this study found was the increased abandonment of the 18–44 age group. Regardless of MUA status, this age group abandoned quality measured prescriptions at nearly double the rate of the 65 and older age group. Most published literature validates this finding that groups younger than 65 are more likely than those over 65 to be non-adherent. One such study saw a very similar outcome where patients between the ages of 18–34 were the most likely to abandon prescriptions while seniors above the age of 65 were 45% less likely.^1^ Another study, involving the discontinuation of statin medications, also saw higher abandonment rates for those 25–49 as compared with those ages of 61–75.^7^ This could be due to lack of insurance, not understanding the importance of the medications or other factors. This is an area that could benefit from future research.

While this study benefitted from its large sample size, there were some limitations. Results were from only one regional division with most pharmacies located in the mid-south region of the U.S. Beyond issues of generalizability, there also exists the possibility that a patient may have abandoned their medication at this specific pharmacy chain, only to pick it up at another. Another limitation of this study was the uneven study groups. Approximately 82% of prescriptions were filled by patients in MUAs while only 18% were not. Moreover, the is a limitation within the medically underserved group itself. During this study, all individuals within areas classified as “medically underserved,” were grouped together. This does not take into account variables, such as, if the area is rural or urban, the patient's ethnicity, race, or socioeconomic status. The data used in this study also could have included, but not accounted for, data for the same patient multiple times, if that patient repeatedly had a medication filled and then abandoned.

## 6 Conclusion

Patients in MUAs abandon quality measured prescriptions at a statistically significant higher rate when compared to patients who live in areas that are not considered to be medically underserved. Moreover, during the COVID-19 pandemic patients living in MUAs had a statistically significant decrease in prescription abandonment while those in non-MUAs did not statistically change. Overall, this study showed that more research needs to be done on interventions to decrease prescription abandonment, especially in MUAs.

## Ethics approval

Research was approved by the University of Tennessee Health Science Center Institutional Review Board (Memphis, TN).

## Funding support

This research did not receive any specific grant from funding agencies in the public, commercial, or not-for-profit sectors.

## CRediT authorship contribution statement

**Minghui Li:** Writing – review & editing, Writing – original draft, Supervision, Software, Methodology, Data curation. **Matthew Harmon:** Writing – review & editing, Writing – original draft, Project administration, Investigation. **Mike Wasson:** Writing – review & editing, Conceptualization. **Lindsey Henson:** Writing – original draft, Resources. **Hunter Hill:** Writing – review & editing, Resources, Conceptualization. **Brad Ian Jobe:** Writing – original draft. **Sydnee E. Hewitt:** Writing – review & editing, Supervision, Resources. **Kenneth C. Hohmeier:** Writing – review & editing, Writing – original draft, Supervision, Resources, Project administration, Methodology, Investigation, Data curation, Conceptualization.

## Declaration of competing interest

The authors declare that they have no known competing financial interests or personal relationships that could have appeared to influence the work reported in this paper.

## Appendix A Medications included in analysis

Oral Anti-Diabetic Medications:

metformin (+/− alogliptin, canagliflozin, dapagliloflozin, empagliflozin, ertugliflozin, glipizide, glyburide, linagliptin, pioglitazone, repaglinide, rosiglitazone, saxagliptin, sitagliptin), chlorpropamide, glimepiride (+/− pioglitazone), glipizide (+/− metformin), glyburide (+/− metformin), tolazamide, tolbutamide, pioglitazone (+/− alogliptin, glimepiride, metformin), rosiglitazone (+/− metformin), alogliptin (+/− metformin, pioglitazone), linagliptin (+/− empagliflozin, metformin), saxagliptin (+/− metformin, dapagliflozin), sitagliptin (+/− metformin, ertugliflozin), nateglinide, repaglinide (+/− metformin), canagliflozin (+/− metformin), dapagliflozin (+/− metformin, saxagliptin), empagliflozin (+/− metformin, linagliptin), ertugliflozin (+/− sitagliptin, metformin).

Renin Angiotensin System (RAS) Antagonists:

aliskiren (+/− hydrochlorothiazide), azilsartan (+/− chlorthalidone), candesartan (+/− hydrochlorothiazide), eprosartan (+/− hydrochlorothiazide), irbesartan (+/− hydrochlorothiazide), losartan (+/− hydrochlorothiazide), olmesartan (+/− amlodipine, hydrochlorothiazide), telmisartan (+/− amlodipine, hydrochlorothiazide) valsartan (+/− amlodipine, hydrochlorothiazide nebivolol), benazepril (+/− amlodipine, hydrochlorothiazide), captopril (+/− hydrochlorothiazide), enalapril (+/− hydrochlorothiazide), fosinopril (+/− hydrochlorothiazide), lisinopril (+/− hydrochlorothiazide), moexipril (+/− hydrochlorothiazide), perindopril (+/− amlodipine), quinapril (+/− hydrochlorothiazide) ramipril.

trandolapril (+/− verapamil), sacubitril/valsartan.

HMG-CoA reductase inhibitors Medications:

atorvastatin (+/− amlodipine), Fluvastatin, lovastatin (+/− niacin), pitavastatin, pravastatin, rosuvastatin.

simvastatin (+/− ezetimibe, niacin).
